# Factors Affecting Domiciliary Non-Invasive Ventilation Compliance

**DOI:** 10.1007/s00408-022-00557-8

**Published:** 2022-07-24

**Authors:** Amar J. Shah, Katia Florman, Nitika Kaushal, Hiu Fung Kwong, Akul Karoshi, Laura White, Ryan Walker, Yan-Pin Lin, Ho Juen Ko, Anita Saigal, Nikesh Devani, Stephanie K. Mansell, Swapna Mandal

**Affiliations:** 1 Present Address: Royal Free Hospital NHS Foundation Trust, Respiratory Medicine, Pond Street, London, NW3 2QG UK; 2 UCL Respiratory, University College London, London, UK

**Keywords:** Compliance, Non-invasive ventilation, Schizophrenia

## Abstract

Few international studies have investigated factors affecting domiciliary non-invasive ventilation (D-NIV) compliance, and data from the UK are limited. We assessed compliance (defined as ≥ 4 h/night for at least 70% of the time) in a retrospective UK population study, at three time points (0–1 month, 3–4 months and 11–12 months), for all patients commenced on D-NIV over a 5-year period. A total of 359 patients were included. Non-compliant vs. compliant patients were significantly younger (median age 64 (IQR 52–72) vs. 67 (58–75) years, *p* = 0.032) and more likely to have schizophrenia, consistent at both 3–4 months (5% vs. 1%, *p* = 0.033) and 11–12 months (5% vs. 2%, *p* = 0.049). Repeated measures ANOVA demonstrated that the minutes [median (IQR)] of D-NIV used significantly increased at the three time points (0–1 month, 3–4 months and 11–12 months) for patients with hypertension [310 (147.5–431) vs. 341 (89–450) vs. 378 (224.5–477.5), *p* = 0.003]; diabetes [296.5 (132.5–417.5) vs. 342.5 (94.5–438.5) vs. 382 (247.5–476.25), *p* = 0.002] and heart failure [293 (177–403) vs. 326 (123–398) vs. 365 (212–493), *p* = 0.04]. In conclusion, younger and comorbid schizophrenic patients have lower D-NIV compliance rates, and our data suggest that persistence with D-NIV over a year may improve overall use.

## Introduction

Domiciliary non-invasive ventilation (D-NIV) is widely accepted as standard of care in patients with chronic hypercapnic respiratory failure caused by chest wall deformity (CWD), neuromuscular disease (NMD), stable hypercapnic chronic obstructive pulmonary disease (COPD) and obesity hypoventilation syndrome (OHS) [1–3]. It is well recognised that D-NIV adherence of more than four hours per day is crucial to improve hypercapnia and patient morbidity and mortality [3–5].

There have been few international studies investigating factors affecting D-NIV compliance, with differing outcomes [6–9]. The largest, from the Netherlands (*n* = 301), found that compliance was higher in patients with amyotrophic lateral sclerosis compared to other conditions and lower in those with anxiety and depression. This study found compliance was not associated with age or other comorbidities [9]. There are a paucity of data from the UK population.

Our aim was to identify factors that impact D-NIV compliance in a UK population.

## Methods

This was a 5-year, cross-sectional, retrospective service evaluation for all patients commenced on D-NIV in a specialist London teaching hospital from 01.01.2014 to 31.12.2019. Patients were all initiated on D-NIV following a portable level 3 home sleep study and a capillary or arterial blood gas (on air). Level 3 sleep studies have shown good diagnostic performance when compared to level 1 gold standard inpatient sleep studies in a previous systematic review and meta-analysis [10]. All patients had face-to-face D-NIV set-up by a respiratory sleep practitioner and a clinic review with a respiratory sleep and ventilation specialist. Patients were given detailed instructions on how to use D-NIV, taught the importance of D-NIV adherence and offered follow-up appointments 3–6 monthly.

As this was a service evaluation, the Health Research Authority, UK state that ethical review is not necessary. Data downloads from the NIV devices were used to check compliance for all D-NIV patients in their first year of use at three distinct time points (0–1 month, 3–4 months, and 11–12 months). Patients were deemed compliant if they had used their NIV for ≥ 4 h/night for at least 70% of the time. We collected patient baseline demographic data including age, gender, ethnicity, deprivation index, body mass index (BMI), smoking history and comorbidities. Patient comorbidities were assessed as per the documented diagnosis in patient clinical records. Relevant comorbidities (cardiovascular and mental health) were included in the statistical analysis. We also collected information on index sleep studies (including apnoea/hypopnoea index (AHI), oxygen desaturation index (ODI), mean saturations and time spent with saturations below 90%), indications for D-NIV, Epworth sleepiness scores, blood gas results and the percentage of time patients had large ventilator leak. The need for informed consent was waived in view of the retrospective nature of the analysis and that only data available in the medical records were collected and studied. Quantitative variables were described as means (± standard deviation) or medians (± interquartile ranges (IQRs)) as appropriate. Categorical data were analysed using Chi-squared test, whilst continuous data were analysed using Mann–Whitney *U* test (non-normally distributed data) or *T* test (normally distributed data). A one-way analysis of variance (ANOVA) with repeated measures was used to compare the means of continuous data at the three distinct time points. A *p*-value of < 0.05 was considered significant.

## Results

A total of 359 patients were commenced on D-NIV across 5 years (median age 65 (IQR 54–72.5) years; 55% male; 62% white; 66% ever smokers; mean nocturnal saturations 86% ± 5.2% and mean PaCO_2_ 6.63 ± 1.48). Table 1 shows the baseline characteristics of our population. In terms of indications for commencing D-NIV: 55% had OHS, 31% COPD, 9% NMD and 5% CWD, with some patients having an overlap of conditions (28%).

**Table 1:** Baseline characteristics of the whole population

Patient characteristics (*n* = 359)	
Age (median, IQR)	65 (54–72.5)
Male (%)	198 (55)
White (%)	224 (62)
Ever-Smokers (%)	181 / 275 (66)
Index of multiple deprivation decile* (Median, IQR)	4 (3–7)
Patient Comorbidities (*n* = 357)	
Hypertension (%)	157 (44)
Type 2 Diabetes mellitus (%)	98 (27)
Ischaemic heart disease (%)	37 (10)
Heart failure (%)	55 (15)
Atrial fibrillation (%)	28 (8)
Cerebrovascular accident (%)	15 (4)
Depression (%)	19 (5)
Schizophrenia (%)	12 (3)
Baseline sleep study data	
Apnoea–hypopnoea index (Median, IQR)	22.8 (6.9–54.1)
Oxygen desaturation index (Median, IQR)	32.3 (10.6–61.5)
Nocturnal oxygen saturations (%mean ± SD)	86.1 ± 5.2
Time spent below 90% (%mean ± SD)	61.2 ± 34.9
PaCO2 kPa (mean ± SD)	6.63 ± 1.48
PaO2 kPa (mean ± SD)	8.37 ± 1.73
Bicarbonate level (mean ± SD)	27.6 ± 4.9
Indications for non-invasive ventilation	
Obesity hypoventilation syndrome (%)	198 (55)
Neuromuscular disease (%)	31 (9)
Chest wall deformity (%)	18 (5)
Chronic obstructive pulmonary disease (%)	110 (31)
Isolated OSA (failed CPAP therapy) (%)	23 (6)
Other** (%)	12 (3)

*Index of multiple deprivation is an official measure of relative deprivation for small areas in England, ranking every small area in England from 1 (most deprived) to 32,844 (least deprived). This is presented as a decile with the 1 being the most deprived and 10 being the least

**Other cases included bronchiectasis, central sleep apnoea and where the cause was unknown

*OSA* obstructive sleep apnoea, *CPAP* continuous positive airway pressure

The percentage of patients who were compliant increased across the three time points [0–1 month: 42%; 3–4 months: 47% and 11–12 months: 53%, *p* < 0.001]. Across all three time points, the average minutes used [median (IQR)] were significantly lower (*p* < 0.001) in the non-compliant vs. compliant groups; [0–1 month 151 (0–253) vs. 430 (371–480); 3-4 months 66 (0–255) vs. 440 (383–509); 11–12 months 40 (0–258) vs. 448 (393–519)].

At 0–1 month, non-compliant vs. compliant patients were significantly younger (median 64 years (52–72) years vs. 67 (58–75) years, *p* = 0.032) and lived in a more deprived area (index of multi-deprivation decile 4 (3–6) vs. 5 (3–7), *p* = 0.013). There were no other significant differences in other baseline demographics including BMI, medical comorbidities, (including hypertension, diabetes, ischaemic heart disease, depression, schizophrenia, heart failure, stroke, and atrial fibrillation), D-NIV indication, Epworth sleepiness score (ESS), index sleep study parameters (AHI, ODI, mean oxygen saturations and time spent with saturations below 90%), blood gas parameters and mask leak.

At 3–4 months, non-compliant vs. compliant patients were significantly younger (median 63 years (51–71) vs. 67 years (58–75), *p* = 0.002), had a lower prevalence of hypertension (39% vs. 50%, *p* = 0.041) and higher prevalence of schizophrenia (5% vs. 1%, *p* = 0.033). There were no other significant differences in other baseline demographics, medical comorbidities, D-NIV indication and index sleep study parameters.

At 11–12 months, non-compliant vs. compliant patients were significantly younger (median 63 years (50–71) vs. 67 years (58–75), *p* = 0.002) and had a higher prevalence of schizophrenia (5% vs. 2%, *p* = 0.049). No other differences were found, and the difference in hypertension seen at 3–4 months was not observed.

**Fig. 1:**
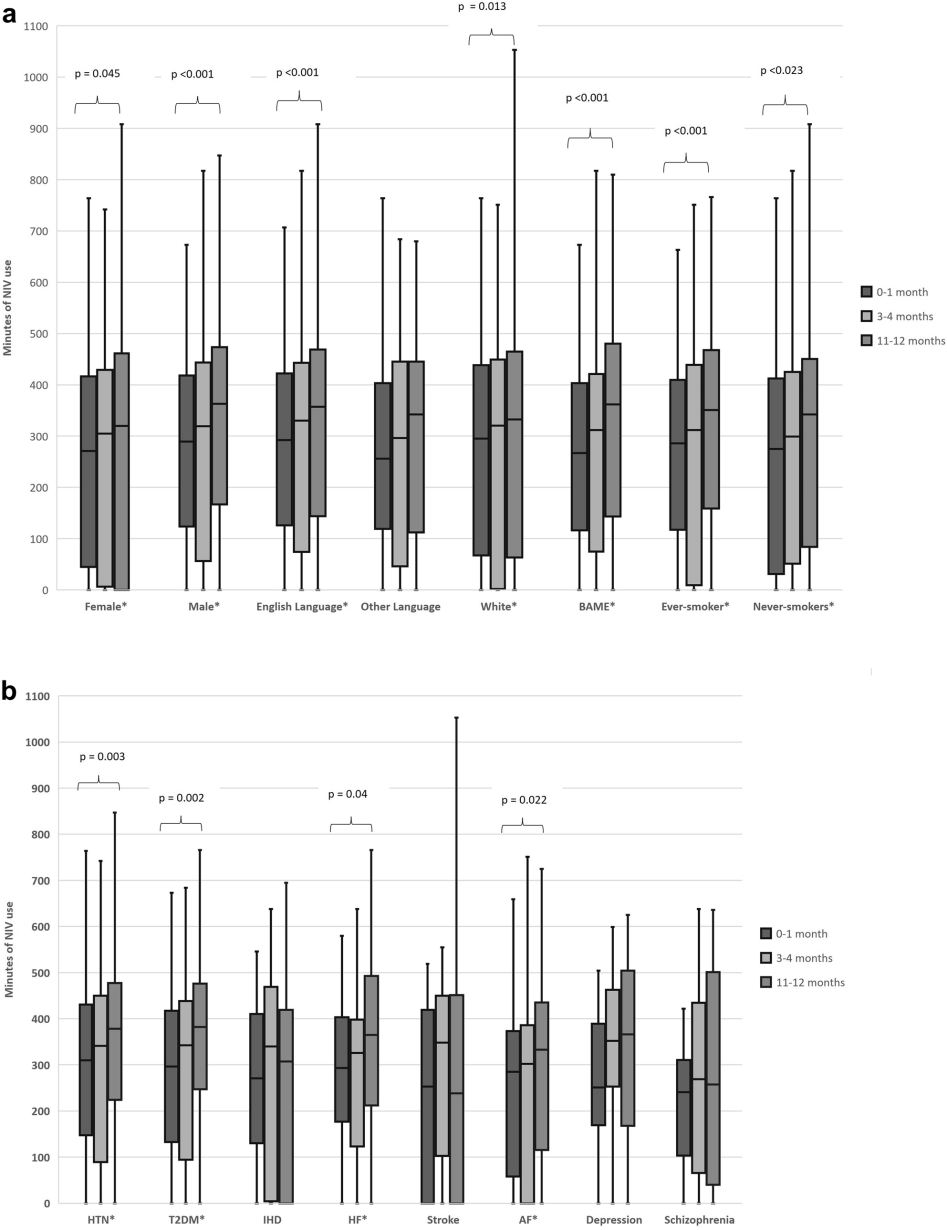

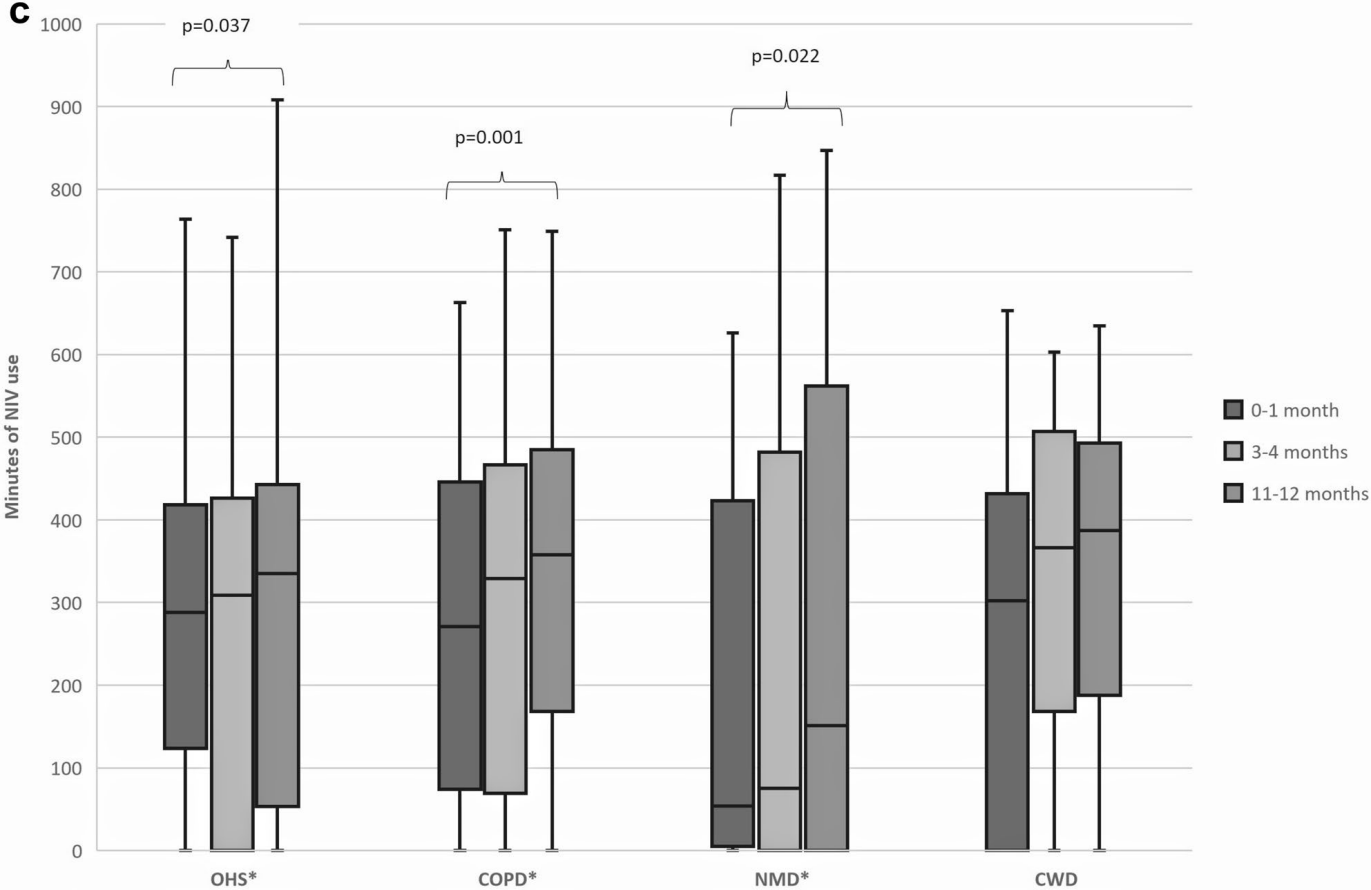
**a** Box-plot of the number of minutes of NIV use across the three time points in different demographic sub-groups of our cohort. **b** Box-plot of the number of minutes of NIV use across the three time points in patients with different commodities. **c** Box-plot of the number of minutes of NIV use across the three time points in patients with different index indications for NIV. The length of the box represents the interquartile range (IQR) with the top of the box representing the third quartile and bottom the first quartile. The width of the box is arbitrary. The horizontal line across the box represents the median value. The whisker length has been determined by calculating the maximal whisker length (1.5 times the IQR). The 3rd quartile plus maximal whisker length denotes the upper boundary, whilst the 1st quartile minus the maximal whisker length denotes the lower boundary. Any values falling outside these boundaries have been classified as outliers and have not been shown. The minimum and maximal values shown are the smallest and largest value in the data set that falls within the set boundaries. This follows standardised nomenclature for a modified box-plot as originally described by Tunkey in 1977. [14] *AF* atrial fibrillation, *BAME* Black, Asian and Minority Ethnic, *COPD* chronic obstructive pulmonary disease, *CWD* chest wall deformity, *HTN* hypertension, *IHD* ischaemic heart disease, *NMD* neuromuscular disease, *OHS* obesity hypoventilation syndrome, *T2DM* type 2 diabetes. All statistical analysis was conducted using a repeat measures ANOVA with a Greenhouse–Geisser correction

Repeated measures ANOVA with a Greenhouse–Geisser correction demonstrated that the minutes of D-NIV used significantly increased at the three time points for different demographics (Fig. 1a) comorbidities (Fig. 1b) and indications for D-NIV (Fig. 1c).

## Discussion

To our knowledge this is the largest population of D-NIV patients to have been studied with a specific view to identify factors affecting compliance. In our cohort, 53% of patients were compliant with NIV at 1 year. Whilst this is a high rate of non-compliance, it includes all patients and is comparable to previously published studies where compliance ranged from 30 to 50%. [6, 7, 9] Throughout the various time points older patients were significantly more likely to be compliant. Additionally, at 3–4 months and 11–12 months, patients with comorbid schizophrenia were more likely to be non-compliant. We did not find the same to be true for patients with comorbid depression, a finding that was previously concluded by a group in the Netherlands. [9] However, this is an important finding that can shape future practice. These findings suggest that within our population younger patients and those with schizophrenia may need more intensive support to improve compliance and engagement with D-NIV. Moreover, patients with schizophrenia did not significantly improve the number of minutes used through the three time points [0–1 month median 241 (IQR 103.25–310.75) vs. 3–4 months 269(65.75–434.75) vs. 11–12 months 257.5 (40–501.25), *p* = 0.49], implying that we may not be providing adequate support for this sub-group of patients. Therefore, collaboration with mental health services may improve D-NIV compliance in these vulnerable individuals. This finding is likely to be applicable in other services. However, it is worth noting that our population had a higher prevalence of schizophrenia (3%) than previously reported work in a similar size NIV population (1%) and national UK prevalence estimates (0.7%). [11, 12] Whilst the reason behind this is not clear, all patients received the same initial patient education, and our data suggest that patients with schizophrenia may need an increased level of support.

There was a significant increase in the number of minutes of D-NIV use across the year when sub-group analysis was performed. This led to an increase in a median 76.5 (range: − 20 to 187) minutes of additional D-NIV use when comparing 0–1 months to 11–12 months. This is clinically relevant [13] and further emphasises the need for persistence and perseverance with therapy encouragement by healthcare practitioners. It also suggests supporting patients for at least a year to optimise compliance.

The main limitation to this service evaluation is the retrospective nature; however, this has enabled analysis of a large number of patients across several different time points. Additionally, there were some missing data due to lack of data card retrieval; however, this was only a small proportion (1%) of our data. Another limitation is that we did not assess ventilator settings other than leak given our hospital uses average volume-assured pressure support ventilation (AVAPS) and so the exact inspiratory pressure and expiratory pressure requirements often vary night to night. We also did not evaluate other factors that may impact patient comfort with D-NIV and therefore compliance, including mask fit, frequency of mask replacement, the actual inspiratory or expiratory pressure requirements and the use of comfort functions, such as ramp.

In conclusion, this service evaluation demonstrates that younger and comorbid schizophrenic patients have lower compliance rates and thus need increased supportive measures to improve compliance. These data also suggest that persistence with NIV over the course of at least a year may improve the average number of minutes that patients use NIV. NIV can improve morbidity, mortality and patient quality of life, thus finding novel ways to engage patients and improve compliance is of utmost importance. Therefore, prospective studies are necessary to identify what tools (e.g. motivational interviewing, psychological therapies) will improve NIV compliance for the betterment of patient care.
